# Catalytic Epoxidation of a Technical Mixture of Methyl Oleate and Methyl Linoleate in Ionic Liquids Using MoO(O_2_)_2_·2QOH (QOH = 8-quinilinol) as Catalyst and NaHCO_3_ as co-Catalyst

**DOI:** 10.3390/molecules14082935

**Published:** 2009-08-10

**Authors:** Shuang-Fei Cai, Li-Sheng Wang, Chuan-Lei Fan

**Affiliations:** School of Chemical Engineering and Environment, Beijing Institute of Technology, Beijing 100081, China

**Keywords:** ionic liquids, epoxidation of vegetable oil, molybdenum, hydrogen peroxide

## Abstract

The oxo-diperoxo molybdenum(VI) complex MoO(O_2_)_2_·2QOH (QOH = 8-quinilinol) was prepared and characterized by elemental analysis, IR and UV-Vis spectra. The ionic liquids (ILs) [bmim][BF_4_], [hydemim][BF_4_], and [bmim][PF_6_] were characterized by ^1^H-NMR and UV-Vis spectra. The epoxidation of a technical mixture of methyl oleate and methyl linoleate with H_2_O_2_, in [bmim][BF_4_], [hydemim][BF_4_] and [bmim][PF_6_], catalyzed by MoO(O_2_)_2_·2QOH (QOH = 8-quinilinol) and with NaHCO_3_ as co-catalyst has been studied for the first time. It was found that high conversions of methyl oleate and methyl linoleate to their respective oxidation products, as well as the total selectivity of their oxidation products to oxirane in [hydemim][BF_4_] were obtained. Also, the IL phases containing the Mo(VI) catalyst can be readily recycled by washing with diethyl ether and drying, and the Mo(VI) catalyst can be reused at least five times.

## 1. Introduction

In recent years, much emphasis has been placed on the utilization of renewable resources as an alternative to petrochemical-based feedstocks. In this context, vegetable oils are undoubtedly promising candidates. Not only they are widespread, inexpensive, eco-compatible, renewable and non-noxious, but, above all, several multi-functionalised molecules can be obtained by their chemical modification. For example, the unsaturated olefinic bonds in vegetable oils can be epoxidized to give epoxy oils, which have been already used widely as PVC-plasticizers and stabilizers, as reactive diluents for paints, as additives in lubricants, etc. [1]

Traditionally, the only commercial source of epoxidized oils is based on the Prileshajev peracid process [2]. This procedure, however, has several drawbacks that require improvement: (i) selectivity to epoxidized products is relatively low due to acid-catalyzed oxirane ring opening side reactions; (ii) the separation of acidic by-products, whose presence may be detrimental for further applications, is not easy; (iii) the handling of highly-concentrated hydrogen peroxide and strong acids is dangerous and causes corrosion problems. For all these reasons there is considerable interest in finding an alternative epoxidation route for these substrates [3,4,5,6].

To this end, efficient metal-catalyzed epoxidations of vegetable oils have been investigated, especially using high-valence catalysts based on titanium [7], tungsten [8] and rhenium [9]. The use in oxidation of molybdenum(VI) catalysts [10] containing the stable oxo-peroxo core [11] is well-established, and with them a variety of organic substrates [12,13,14], including alkenes, alcohols, amides and nitro compounds, can be catalytically oxidized under homogeneous as well as heterogeneous conditions [15]. Recently, several interesting oxo-peroxo molybdenum(VI) complexes such as [MoO(O_2_)_2_L_2_] (L = 8-quinilinol) [16], have proven to be excellent room temperature (rt) catalysts for the epoxidation using the H_2_O_2_/NaHCO_3_ system of many alkenes such as styrene, 1-octene, etc. in CH_3_CN solutions. Not only is this catalyst-oxidant system economical and the reaction conditions mild, but hydrogen peroxide is a readily-available and green oxidant [17], so, it would be undoubtedly noteworthy if this catalytic system could also be used in the epoxidation of vegetable oils, an area in which to our knowledge no work has been conducted. 

Furthermore, increased awareness in recent years of the detrimental environmental effects of organic solvents has resulted in rapid growth of research on alternative reaction media, and more attention has been given to the reusability of solvents and catalysts for the development of cost-effective protocols. Ionic liquids (ILs) are a promising alternative to classical solvents because ILs, especially those based on 1,3-dialkylimidazolium cations, such as 1-*n*-butyl-3-methylimidazolium tetrafluoroborate ([bmim][BF_4_]) and its hexafluorophosphate analog ([bmim][PF_6_]), are air and moisture stable, and possess negligible vapor pressure, low viscosity, high thermal and chemical stability and a wide electrochemical window [18,19,20,21]. They can also be recycled in many cases. Various classical reactions such as Friedel-Crafts [22], Diels-Alder [23], alkylation [24], hydrogenation [25], Heck [26], hydroformylation [27] and oligomerisation [28] using ILs as solvents have been reported. They have also emerged as ideal immobilization media for catalysts, which combine the advantages of ILs and heterogeneous supports, such as increased reaction rates, enhanced catalytic efficiency, simplified product separation together with improved catalyst recyclability, therefore, the use of ILs as solvents in organic reactions has become a particular focus of research [29].

To our knowledge, however, there is no literature describing the catalytic epoxidation of vegetable oils using ILs as reaction media/immobilization media for transition metal complex catalysts. In this paper, we report the epoxidation of a technical mixture of methyl oleate and methyl linoleate with H_2_O_2_, catalyzed by [MoO(O_2_)_2_·2QOH] (QOH = 8-quinilinol) and NaHCO_3_ as a co-catalyst. For comparison, the ILs [bmim][BF_4_], [hydemim][BF_4_], and [bmim][PF_6_] were used as solvents, respectively.

## 2. Results and Discussion

### 2.1. Catalyst preparation

**Scheme 1:**
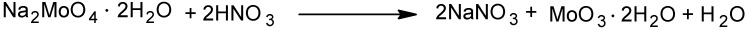
The preparation of MoO_3_·2H_2_O.

**Scheme 2:**
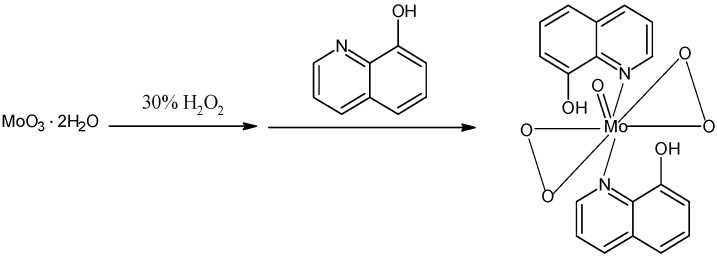
The preparation of di-8-quinolinolate [MoO(O_2_)_2_·2QOH] (QOH = 8-quinilinol).

Briefly, MoO_3_·2H_2_O, prepared according to a published procedure [30] (Scheme 1) was dissolved in 30% H_2_O_2_ and mixed with an acetic acid solution of 8-quinolinol to give the [MoO(O_2_)_2_·2QOH] (QOH = 8-quinilinol) catalyst (Scheme 2), which was characterized by elemental analysis, IR and UV spectroscopy. 

### 2.2. Catalytic performance

#### 2.2.1. UV-Vis spectral study of [MoO(O_2_)_2_·2QOH] (QOH = 8-quinilinol) alone

**Figure 1:**
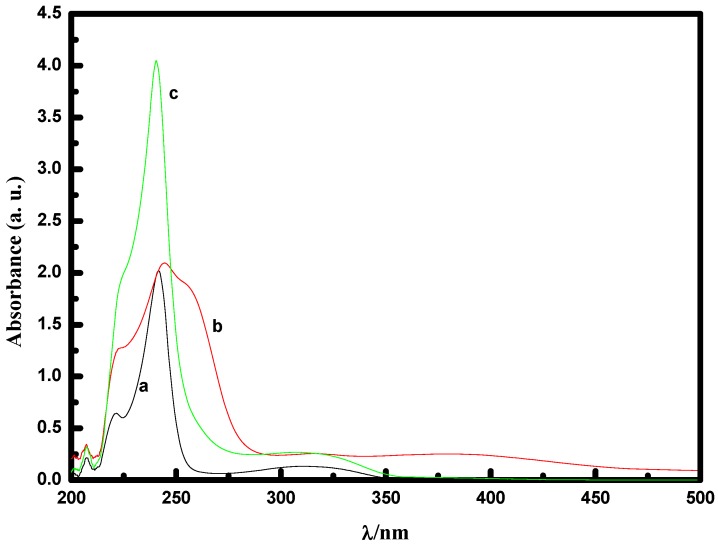
The UV-Vis spectra of 8-quinilinol (curve a), [MoO(O_2_)_2_·2QOH] (QOH = 8-quinilinol) (curve b), [MoO(O_2_)_2_·2QOH] (QOH = 8-quinilinol) + H_2_O_2_ + substrate (curve c).

The UV-Vis spectra of 8-quinilinol in CH_2_Cl_2_ displayed two shoulders at 242 and 311 nm (Figure 1, curve a), while the oxo-diperoxo molybdenum(VI) complex [MoO(O_2_)_2_·2QOH] (QOH = 8-quinilinol) showed two strong absorption bands at 244 and 380 nm (Figure 1, curve b), and a small absorption band at 315 nm. Further, it showed a very broad low intensity absorption ranging from 300 to 500 nm. The 380 nm absorption band could be assigned to the ligand-to-metal charge transfer [16]. [MoO(O_2_)_2_·2QOH] (QOH = 8-quinilinol) was found to be slightly soluble in the ILs used, but almost immiscible with substrate at rt. When hydrogen peroxide is added, interestingly, it dissolves immediately and gives an orange solution. To find out more about the mechanism of reaction and confirm the nature of the reactive intermediate, the UV-Vis spectrum of the reaction mixture resulting from the addition of substrate to a solution of catalyst and hydrogen peroxide was studied. Two strong absorption bands at 240 and 308 nm (Figure 1, curve c) were observed. Similarly, a small absorption band was found at 362 nm. Additionally, compared with [MoO(O_2_)_2_·2QOH] (QOH = 8-quinilinol), the absorbance of the peak in the 300 to 500 nm region was decreased considerably. It is believed that the new small peak appearing at 362 nm (lit. [31] 364 nm), corresponds to the active oxo-dioxo molybdenum species which has been proposed as the catalyst precursor [32], and is generated from the oxo-diperoxo molybdenum species. Each double bond can be epoxidized in the presence of monooxodiperoxo species, and the intermediate oxo-dioxo species formed regenerates the monooxodiperoxo centers.

#### 2.2.2. Effect of NaHCO_3_ as a co-catalyst

**Scheme 3:**
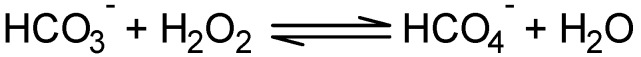
The production of HCO_4_^-^.

Table 1 shows the results of epoxidation of methyl oleate and methyl linoleate. In all the cases, the conversion of the former to its oxidation products was higher than that of the latter, a result of the difference in the number of double bonds in these two unsaturated esters. Without any added solvent the total conversion of methyl oleate and methyl linoleate to their oxidation products was low (41%, Entry 1), and with the use of only NaHCO_3_ without catalyst, no conversion of methyl linoleate or methyl linoleate was observed (Entry 2). Further, the catalytic efficiency when H_2_O_2_ is used as a sole oxidant is poor (Entries 6 and 7). When NaHCO_3_ is used together with H_2_O_2_, however, the efficiency of the system is greatly increased. The key aspect of such a reaction is that H_2_O_2_ and NaHCO_3_ react in an equilibrium process to produce peroxymonocarbonate HCO_4_^-^ which is a more reactive nucleophile than H_2_O_2_ and speeds up the epoxidation reaction (Scheme 3) [33,34].

**Table 1:** Epoxidation of methyl oleate and methyl linoleate with hydrogen peroxide catalyzed by [MoO(O_2_)_2_·2QOH] (QOH = 8-quinilinol) in ILs.^a^

**Entry**	**Solvent**	**Conversion (%)**			**Selectivity (%) ^e^**	**TON(TOF) ^f^**
		methyl oleate ^b^	methyl linoleate ^c^	total ^d^		
1	no solvent	55	31	41	90	3690(1845)
2	no catalyst	0	0	0	0	0
3	[bmim]BF_4_	92	78	84	93	7812(3906)
4	[bmim]PF_6_	75	44	57	94	5358(2679)
5	[Hydemim]BF_4_	96	89	92	95	8740(4370)
6	[bmim]PF_6_^g^	60	32	44	92	4048(2024)
7	[Hydemim]BF_4_^g^	84	54	67	94	6298(3149)
8	CH_3_CN	85	63	72	92	6624(3312)
9	30% CH_3_CN + 70% [hydemim]BF_4_	94	84	88	95	8360(4180)
10	C_2_H_5_OH	81	45	60	93	5580(2790)
11	30 % C_2_H_5_OH + 70% [hydemim]BF_4_	90	74	81	95	7695(3848)

^a^ Reaction conditions: [M_c=c_:H_2_O_2_:catalyst:NaHCO_3_], 1:4:0.0001:0.3; M_c=c_ was the total amount (mmol) of c=c and calculated by M_c=c_ = M_methyl oleate_ + 2 M_methyl linoleate_; V_solvent_, 2 mL; T, 303 K; t, 2 h. ^b^ The relative percentage conversion of methyl oleate to oxidation products, was calculated by expression (3) (see Appendix). ^c^ The relative percentage conversion of methyl linoleate to oxidation products, was calculated by expression (4) (see Appendix). ^d^ Total conversion of double bonds of methyl oleate and methyl linoleate to the oxidation products, was calculated by expression (10) (see Appendix). ^e^ Total Selectivity of oxidation products to the epoxidized methyl oleate and methyl linoleate, was calculated by expression (16) (see Appendix). ^f^ TON was defined as the ratio of the number of mol of product obtained to the number of mol of catalyst used. TOF was calculated by the expression of TON·time (h^-1^). ^g^ This is the control experiment, excluding the catalyst, but not NaHCO_3_.

#### 2.2.3. Effect of ILs

Both [bmim][BF_4_]- and [hydemim][BF_4_]-containing systems showed higher activity (84% in Entry 3 and 92% in Entry 5, respectively) than the [bmim][PF_6_]-containing system (57 %, Entry 4), which suggested the hydrophobic-hydrophilic properties of the ILs may play a key role in the epoxidation processes of methyl oleate and methyl linoleate. As a comparison with ILs, when using instead organic solvents, including CH_3_CN and C_2_H_5_OH, relatively lower total conversions of methyl oleate and methyl linoleate were obtained (72%, Entry 8 and 60%, Entry 10, respectively). In order to illustrate the effect of ILs more clearly, a mixed solvent of [hydemim][BF_4_] and CH_3_CN or C_2_H_5_OH was used. When 1.4 mL of [hydemim][BF_4_] was added to 0.6 mL of CH_3_CN or C_2_H_5_OH to form a homogeneous solution, about 88 and 81% of methyl oleate and methyl linoleate was converted, respectively (Entries 9 and 11), which suggested the catalytic activity was enhanced by the use of [hydemim][BF_4_].

### 2.3. Recycling of the catalyst

Recycling of the[MoO(O_2_)_2_·2QOH] in the epoxidation of methyl oleate and methyl linoleate was examined with [hydemim][BF_4_] as solvent (Table 2). As shown, the Mo(VI) catalyst immobilized in [hydemim][BF_4_] can be reused at least up to five cycles. The slightly decrease in conversion observed might result from some leaching of [hydemim][BF_4_] into the diethyl ether during the washing. 

**Table 2:** The recycling experiment for the epoxidation of methyl oleate using H_2_O_2_ as oxidant catalyzed by [MoO(O_2_)_2_·2QOH] (QOH = 8-quinilinol) and NaHCO_3_ as co-catalyst in [hydemim][BF_4_]^a^ (^a-e^ same as in Table 1).

Run		1	2	3	4	5
**Conversion (%)**	methyl oleate ^b^	96	92	89	89	87
	methyl linoleate ^c^	89	87	86	82	82
	total ^d^	92	89	87	85	84
**Selectivity (%) ^e^**		95	95	95	95	95

## 3. Experimental

### 3.1. Materials

**Figure 2:**
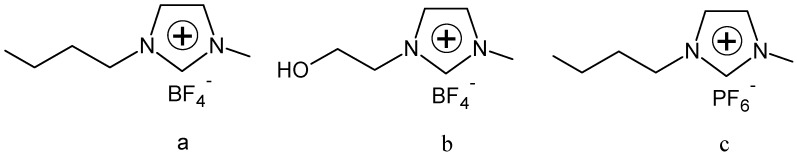
The structures of the ILs [bmim][BF_4_] (a), [hydemim][BF_4_] (b) and [bmim][PF_6_] (c) .

The ILs [bmim][BF_4_], [hydemim][BF_4_], and [bmim][PF_6_] (their structures are shown in Figure 2) were purchased from the Shanghai Chengjie Chemical Co., Ltd, and the mass fraction purities were all above 99%. The mass fraction of water in these ILs was less than 1 × 10^3^ ppm. Fatty acid methyl esters (C. P.) was purchased from the Beijing Jinlong Chemical Co., Ltd., and contained methyl oleate (28.26%), methyl linoleate (19.43%), methyl palmitate (32.69%), methyl octadecanoate (7.28%), methyl dodecanoate (7.05%), methyl tetradecanoate (4.29%), *iso*-propyl myristate (0.53%) and decanoic acid 2-ethylhexyl ester (0.47%) according to GC-MS analysis. Standard samples of methyl oleate (100%, GC-MS) and methyl linoleate (99%) were purchased from Alfa Aesar and Sigma, respectively. Hydrogen peroxide (G. R., 30%) and the other reagents (A. R., >95%) were all provided by the Beijing Chemical Reagents Company and used without further purification.

### 3.2. Instruments

Chemical analyses for C, H, and N were performed by elemental microanalysis (Elementar Vario EL analyzer). IR spectra were measured on a Nicolet MAGNA 750 instrument fitted with a Nic-plan IR microscope. The electronic spectra were recorded on a 756 PC UV/VIS spectrometer. ^1^H NMR spectra were recorded on a Varian Unity Inova-400 spectrometer. The reactions mixtures and their respective fatty acid methyl ester contents were analyzed by GC-MS [DSQ (Thermo Fisher)] with a capillary column (DB-5, 30 m × 0.25 µm × 0.25 mm), equipped with an FID detector. Oven program: 100 °C for 2 min, 5 °C/min to 300 °C and held for 10 min. Both the injector port temperature and the detector transfer line temperature were 250 °C, and helium was used as carrier gas, l mL/min. Spilt sampling, split ratio was 50:1. Sample volume was 0.1 µL. MS: electron ionization, 70 eV. The reaction products were analyzed on a GC2010 with a capillary column (AB-1, 30 m × 0.25 µm × 0.25 mm), equipped with an automatic sampler (AOC-20i) and an FID detector. Oven program: 100 °C for 2 min, 5 °C/min to 300 °C and held for 10 min. Both the injector port temperature and the detector transfer line temperature were 250 °C, and helium was used as carrier gas, 2 mL/min. Spilt sampling, split ratio was 50:1. Sample volume was 2 µL.

### 3.3. Preparation

#### 3.3.1. Preparation and characterization of MoO_3_·2H_2_O

MoO_3_·2H_2_O was prepared according to the published procedure [30] (Scheme 1). Na_2_MoO_4_·2H_2_O (50 g) was dissolved in H_2_O (100 mL) and added to 5 N HNO_3_ (300 mL) at rt. The precipitate was filtered, washed with 4 N HNO_3_ (100 mL) and H_2_O (100 mL), and dried at rt. Yield 25 g, yellow powder (68 %). IR (cm^−1^): 3,520, 3,406, 3,223, 1,623, 968, 905, 744; UV-Vis λ_max_ (nm): 219.40 (ε = 5,836 M^-1^ cm^-1^).

#### 3.3.2. Preparation and characterization of [MoO(O_2_)_2_·2QOH] (QOH = 8-quinilinol)

[MoO(O_2_)_2_·2QOH] (QOH = 8-quinilinol) was prepared based on the published procedure (Scheme 2) [16]. An aqueous solution (25 mL) of MoO_3_·2H_2_O (1.25 g; 6.0 mmol) was dissolved in 30 % (w/v) H_2_O_2_ (40 mL; 13.9 mmol) by stirring at rt to give a pale yellow solution. Addition of 8-quinolinol (2.02 g; 13.9 mmol) dissolved in 4 M acetic acid (10 mL) to the above solution with stirring (about 10 min) gave a yellow solid. It was filtered off, washed with water, 95% ethanol and finally with diethyl ether and dried under vacuum. Yield: 2.68 g (83%). Found, C, 46.21; H, 2.99; N, 6.21. Calc. for C_18_H_14_N_2_O_7_Mo: C, 46.35; H, 3.00 and N, 6.01%. IR (cm^−1^): 1,620 (w), 1,594 (w), 1,577 (w), 1,558 (m), 1,497 (s), 1,467 [s; v(C-N)], 1,419 (w), 1,406 (w), 1,375 (s), 1,321 (s), 1,306 (w), 1,271 (m), 1,225 (w), 1,210 (w), 1,175 (w), 1,143 (w), 1,107 [s; v(C-O)], 1,062 (w), 1,029 (w), 956 [s; v(Mo=O)], 914 (w), 852 [s; v(O-O)], 820 (s), 749 (s); UV-Vis λ_max_ (nm): 244, 315 (sh), 380 (ε = 4,806 M^-1^ cm^‑1^).

#### 3.3.3. Characterization of the ILs [bmim][BF_4_], [hydemim][BF_4_], and [bmim][PF_6_]

^1^H-NMR: 0.877–0.914 (3H), 1.234–1.290 (2H), 1.732–1.805 (2H), 3.846 (3H), 4.139–4.175 (2H), 7.654 (1H) and 7.720–7.724 (1H) for [bmim][BF_4_] (CDCl_3_); d = 3.98 (3H), 3.99–4.02 (2H), 4.37–4.40 (2H), 4.79 (1H), 7.52 (1H), 7.57 (1H) and 8.77 (1H) for [hydemim][BF_4_] (D_2_O); 0.927–0.964 (3H), 1.357–1.413 (2H), 1.906–1.944 (2H), 4.038 (3H), 4.327–4.363 (2H), 7.673–7.727 (2H) and 8.929 (1H) for [bmim][PF_6_] (CD_3_COCD_3_). UV-Vis (in C_2_H_5_OH): λ_max_ (nm) = 211.60 (ε = 4,403 M^-1^ cm^-1^) for [bmim][BF_4_]; λ_max_ (nm) = 210.95 (ε = 4,109 M^-1^ cm^-1^) for [hydemim][BF_4_]; λ_max_ (nm) = 211.10 (ε = 3,957 M^-1^ cm^-1^) for [bmim][PF_6_].

### 3.4. Epoxidation

Catalyst (0.265 µmol) and NaHCO_3_ (7.950 mmol) were placed in a 50 mL round bottomed flask equipped with a magnetic stir bar. The catalyst was dissolved in the IL (2 mL) by heating and then cooled to rt. To this substrate (ca. 11.66 g) was added and the the reaction mixture stirred at 30 °C. To maintain a constant flow of peroxide during the entire period of reaction, 30 % H_2_O_2_ (106 mmol) was added droppedwise periodically over 2 h, then the reaction mixture was cooled to rt and extracted with diethyl ether (3 × 20 mL). The diethyl ether layers were combined and concentrated under reduced pressure, and a sample was taken for GC analysis and measurement of oxirane oxygen (Appendix). The IL phase including the catalyst was washed with diethyl ether, and dried prior to being recycled.

## 4. Conclusions

The oxo-diperoxo molybdenum(VI) complex MoO(O_2_)_2_·2QOH (QOH = 8-quinilinol) was prepared, and characterized by its elemental analysis, IR and UV-Vis spectra. The ILs [bmim][BF_4_], [hydemim][BF_4_], and [bmim][PF_6_] were characterized by their ^1^H-NMR and UV-Vis spectra. Furthermore, the epoxidation of a technical mixture of methyl oleate and methyl linoleate with H_2_O_2_, catalyzed by MoO(O_2_)_2_·2QOH (QOH = 8-quinilinol) and NaHCO_3_ as a co-catalyst in the ILs [bmim][BF_4_], [hydemim][BF_4_] and [bmim][PF_6_] has been reported in this paper for the first time. High conversions of methyl oleate and methyl linoleate, as well as the total selectivity of their respective oxidation products in [hydemim][BF_4_] were obtained. The IL phase including the Mo(VI) catalyst can be readily recycled by washing with diethyl ether and drying, and the Mo(VI) catalyst can be reused at least five times.

## Appendix: Analysis of products

### Determination of the conversion of methyl oleate or methyl linoleate

The relative percentage conversion of methyl oleate or methyl linoleate to its oxidation products, C_1_ or C_2_, can be calculated by the following expressions:

(1) $${\text{C}}_{1}=(1-\frac{{\text{m}}_{1}^{\prime }}{{\text{m}}_{1}})\times 100$$

(2) $${\text{C}}_{2}=(1-\frac{{\text{m}}_{2}^{\prime }}{{\text{m}}_{2}})\times 100$$

where m_1_’ and m_2_’ are the content of methyl oleate and methyl linoleate unconverted after the epoxidation reaction, respectively. By comparing the retention times of their standard samples with the reaction products, both the content of methyl oleate and methyl linoleate can be determined by GC, using the area normalization method. In addition, m_1_ and m_2_ are the initial content of methyl oleate and methyl linoleate in the substrate, respectively. They were known by GC-MS analysis (m_1_ = 28.26% and m_2_ = 19.43%, respectively).

So:

(3) $${\text{C}}_{1}=(1-\frac{{\text{m}}_{1}^{\prime }}{28.26\%})\times 100$$

(4) $${\text{C}}_{2}=(1-\frac{{\text{m}}_{2}^{\prime }}{19.43\%})\times 100$$

Since the number of double bonds for each mole of methyl oleate is one, while for methyl linoleate it is two, the total conversion of all the double bonds of these two esters, C_t_, can be calculated by the following expression:

(5) $${\text{C}}_{t}=\frac{{\text{M}}_{1}^{\prime }+{2\text{M}}_{2}^{\prime }}{{\text{M}}_{1}+{2\text{M}}_{2}}\times 100$$

where M_1_’, M_2_’ are the amount of methyl oleate and methyl linoleate converted (mol), respectively. M_1_ and M_2_ are the initial amount of methyl oleate and methyl linoleate (mol), respectively. They can be calculated respectively by the following four expressions:

(6) $${\text{M}}_{1}^{\prime }=\frac{{\text{m}}_{\text{sample}}({\text{m}}_{1}-{\text{m}}_{1}^{\prime })}{296.50}$$

(7) $${\text{M}}_{2}^{\prime }=\frac{{\text{m}}_{\text{sample}}({\text{m}}_{2}-{\text{m}}_{2}^{\prime })}{294.50}$$

(8) $${\text{M}}_{1}=\frac{{\text{m}}_{\text{sample}}\cdot {\text{m}}_{1}}{296.50}\cdot$$

(9) $${\text{M}}_{2}=\frac{{\text{m}}_{\text{sample}}\cdot {\text{m}}_{2}}{294.50}\cdot$$

where *m*_sample_ is the mass of the sample measured (g).

So:

(10) $${\text{C}}_{t}=(1-\frac{147.25{\text{m}}_{1}^{\prime }+296.50{\text{m}}_{2}^{\prime }}{99.24})\times 100$$

### Determination of the total selectivity of oxidation products to epoxidized methyl oleate and methyl linoleate

The total yield of the different epoxides can be known by measuring the epoxy value (E) of reaction product, according to the hydrochloric acid – acetone method [35]. E is defined as the content of oxirane oxygen in a sample measured (g/100 g sample):

(11) $$\text{E}=\frac{{\text{m}}_{\text{oxygen}}}{{\text{m}}_{\text{sample}}}\times 100$$

The total percentage yield to oxirane, i.e., the total selectivity of reaction product to oxirane (S_t_), can be calculated by following expression:

(12) $${\text{S}}_{t}=\frac{{\text{E}}_{e}}{{\text{E}}_{c}}\times 100$$

where E_e_ and E_c_ are the experimentally obtained oxirane oxygen and the maximum oxirane oxygen calculated, respectively. E_e_ can be obtained by the following expression:

(13) $${\text{E}}_{e}=\frac{[\text{V}-({\text{V}}_{1}-\frac{{\text{V}}_{2}}{\text{G}}\times {\text{m}}_{\text{sample}})]\text{N}\times 0.016}{{\text{m}}_{\text{sample}}}\times 100$$

where V is the volume of sodium hydroxide standard solution consumed (mL) for measuring the oxirane oxygen of the sample; V_1_ is the volume of sodium hydroxide standard solution consumed (mL) in the blank test for determinating the oxirane oxygen; V_2_ is the volume of sodium hydroxide standard solution consumed (mL) for determinating the acid value of the sample; G is the mass of sample for determinating the acid value (g); N is the concentration of sodium hydroxide standard solution, mol/L; 0.016 is milligram equivalent of oxygen.

The acid value of the sample, X (mg/g sample), is defined as the mass of potassium hydroxide consumed in neutralizing 1 g of sample, and determined by the addition of potassium hydroxide-ethanol standard solution to the sample [36]:

(14) $$\text{X}=\frac{\text{C}({\text{V}}_{2}-{\text{V}}_{0})\times {\text{M}}_{\text{KOH}}}{\text{G}}$$

where C is the concentration of potassium hydroxide-ethanol standard solution (mol/L); V_2_ is the volume of potassium hydroxide-ethanol standard solution consumed (mL) for determinating the acid value of the sample; V_0_ is the volume of potassium hydroxide-ethanol standard solution consumed (mL) in the blank test for determinating the acid value of the sample; M_KOH_ is the molecular mass of potassium hydroxide, 56.11 g/mol.

Since the molecular weight of the oxygen in each mol of epoxidized methyl oleate is 16, while that in epoxidized methyl linoleate is 32, E_c_ can be calculated by the following expression:

(15) $${\text{E}}_{c}=\frac{{\text{M}}_{1}\cdot {\text{C}}_{1}\cdot 16+{\text{M}}_{2}\cdot {\text{C}}_{2}\cdot 32}{{\text{m}}_{\text{sample}}}\times 100$$

Therefore:

(16) $${\text{S}}_{t}=\frac{{\text{E}}_{e}}{0.0152{\text{C}}_{1}+0.0211{\text{C}}_{2}}\times 100$$
